# Enhancing Data Linkage to Break the Chain of COVID-19 Spread: The Taiwan Experience

**DOI:** 10.2196/24294

**Published:** 2021-05-07

**Authors:** Wei-Cheng Lo, Fu-Chung Wang, Li-Yin Lin, Hong-Wei Jyan, Hsuan-Chien Wu, Yao-Liang Huang, I-Ming Parng, Hung-Yi Chiou

**Affiliations:** 1 Master Program in Applied Epidemiology, College of Public Health Taipei Medical University Taipei Taiwan; 2 Department of Information Management Ministry of Health and Welfare Taipei Taiwan; 3 College of Health Technology National Taipei University of Nursing and Health Sciences Taipei Taiwan; 4 Institute of Population Health Sciences National Health Research Institutes Zhunan, Miaoli County Taiwan; 5 School of Public Health, College of Public Health Taipei Medical University Taipei Taiwan; 6 Department of Cyber Security Executive Yuan Taipei Taiwan; 7 Centers for Disease Control Ministry of Health and Welfare Taipei Taiwan; 8 National Immigration Agency Ministry of the Interior Taipei Taiwan

**Keywords:** COVID-19, data linkage, digital health, digital technology, infectious disease, management, National Health Insurance System, prevention, spread, Taiwan

## Abstract

Digital technology has been widely used in health care systems and disease management, as well as in controlling the spread of COVID-19. As one of the most successful countries in combating the COVID-19 pandemic, Taiwan has successfully used digital technology to strengthen its efforts in controlling the COVID-19 pandemic. Taiwan has a well-established National Health Insurance System (NHIS), which provides a great opportunity to develop a nationwide data linkage model in an agile manner. Here we provide an overview of the application of data linkage models for strategies in combating COVID-19 in Taiwan, including NHIS centralized data linkage systems and “from border to community” information-driven data linkage systems during the COVID-19 pandemic. Furthermore, we discuss the dual role of digital technologies in being an “enabler” and a “driver” in early disease prevention. Lastly, Taiwan’s experience in applying digital technology to enhance the control of COVID-19 potentially highlights lessons learned and opportunities for other countries to handle the COVID-19 situation better.

## Introduction

Worldwide, there have been 60 million confirmed cases of COVID-19 and 1.4 million confirmed deaths as of November 30, 2020, and the numbers are still rising to date. When China reported its first COVID-19 outbreak, an early study conducted by Johns Hopkins University in January had forecasted the number of imported cases arriving at each airport inside and outside mainland China, and reported that Taiwan would be the second most affected country by COVID-19 owing to the close economic and transportation links with mainland China [1]. However, Taiwan has defied those expectations. Until November 30, 2020, Taiwan reported only 679 COVID-19 cases, 7 confirmed deaths, and only a 1.03% case fatality rate. Taiwan has been performing well in combating COVID-19 thus far; this may be attributed to the hard lessons learned from the severe acute respiratory syndrome (SARS) outbreak in 2003. The SARS experience had prepared the government and citizens of Taiwan to respond to the COVID-19 pandemic more promptly and cautiously. Countries with a prior experience with SARS prevention (with over 100 confirmed cases and a >10% case fatality rate) also performed well in combating the current COVID-19 outbreak [2,3].

During the SARS outbreak of 2003 in Taiwan, the infected persons did not disclose their history of travel to outbreak areas in order to escape the quarantine; this was a loophole in border control. Furthermore, there was no regulation to prevent potentially infected people from entering hospitals without declaring their risk, resulting in the shutdown of a regional hospital and partial service closure at a medical center [4,5]. Furthermore, the face mask supplies in the market decreased and caused panic related to the purchase of face masks in the community. The devastating experience with the SARS outbreak had led Taiwan to be extremely cautious when China initially announced its COVID-19 outbreak on January 20, 2020. Taiwan quickly established the Central Epidemic Command Center (CECC). The well-developed infrastructure of the CECC has allowed for quick strategic planning, epidemic analysis, and disease prevention, as well as unifying medical resources to successfully contain the outbreak and minimize infections [6]. Meanwhile, digital technology (DT) has played an essential role in combating the COVID-19 pandemic (especially considering its dual roles as an “enabler” and a “driver”) and provided tremendous help in containing SARS-CoV-2, which was not well-exploited during the SARS pandemic.

During the current COVID-19 pandemic, all countries worldwide deployed public health strategies including border control, control of community transmission, enhancement of personal hygiene, and prevention of nosocomial infection, and Taiwan was no exception. Here we elucidate how data linkage was used to strengthen disease prevention and to explore the role of DT in public health and disease control strategies. Based on Taiwan’s successful experience with COVID-19 control, 2 major data linkage models are proposed herein for COVID-19 control. Furthermore, we have discussed the application of data linkage models.

## Applications of the Current National Health Insurance System to Enhance COVID-19 Prevention and Control

**Figure 1:**
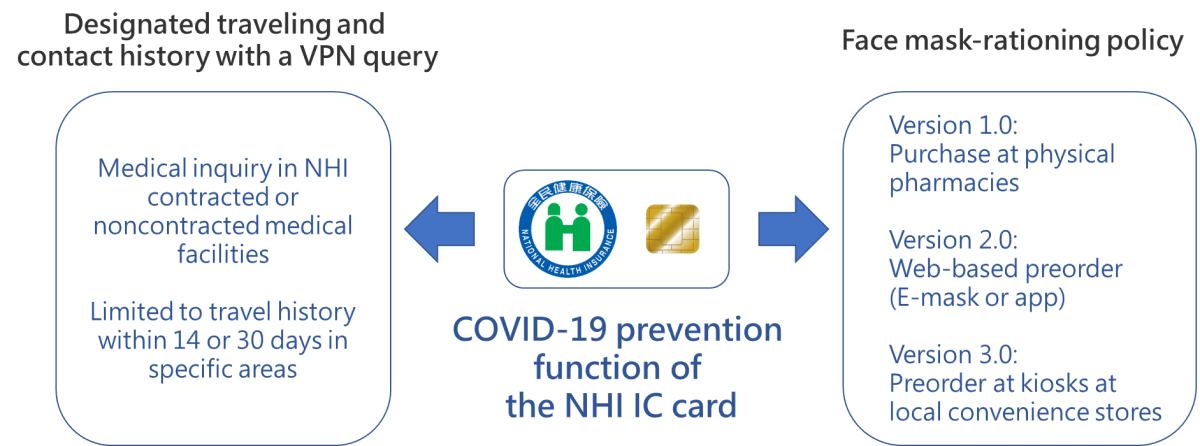
Diagrammatic representation of the NHI IC card usage in COVID-19 prevention policies. NHI: National Health Insurance, VPN: virtual private network.

Taiwan’s National Health Insurance System (NHIS) was initially rolled out in 1995, with the National Health Insurance Administration (NHIA) being the only insurer. Since the NHIS was completely information-driven, 90% of the hospitals and clinics had used the electronic reporting systems to claim medical expenses. Currently, these services are fully automated and have been running smoothly for years. For security reasons, a virtual private network is used to strengthen data security while the information is being processed on the internet. Once the NHIA had collected, reported, and complied all medical expenses into the “centralized database,” big data were used to automatically review and process all medical claims received from hospitals and clinics. As a benefit, Taiwan’s NHIS has the lowest administrative cost worldwide [7,8]. In addition, NHIA issues National Health Insurance IC cards (NHI IC cards) as an insurance certificate. In general, Taiwanese citizens present the NHI IC card during medical visits. The NHI IC card enables physicians to obtain the most recent medical records of the patient. Moreover, detailed past medical records and medication use can also be accessed by linking this information with a medical cloud information exchange system, the MediCloud. Under the NHIS, two important pandemic prevention features were established: travel history tracking and the mask-rationing plan for purchase (Figure 1).

### Travel History Tracking

**Figure 2:**
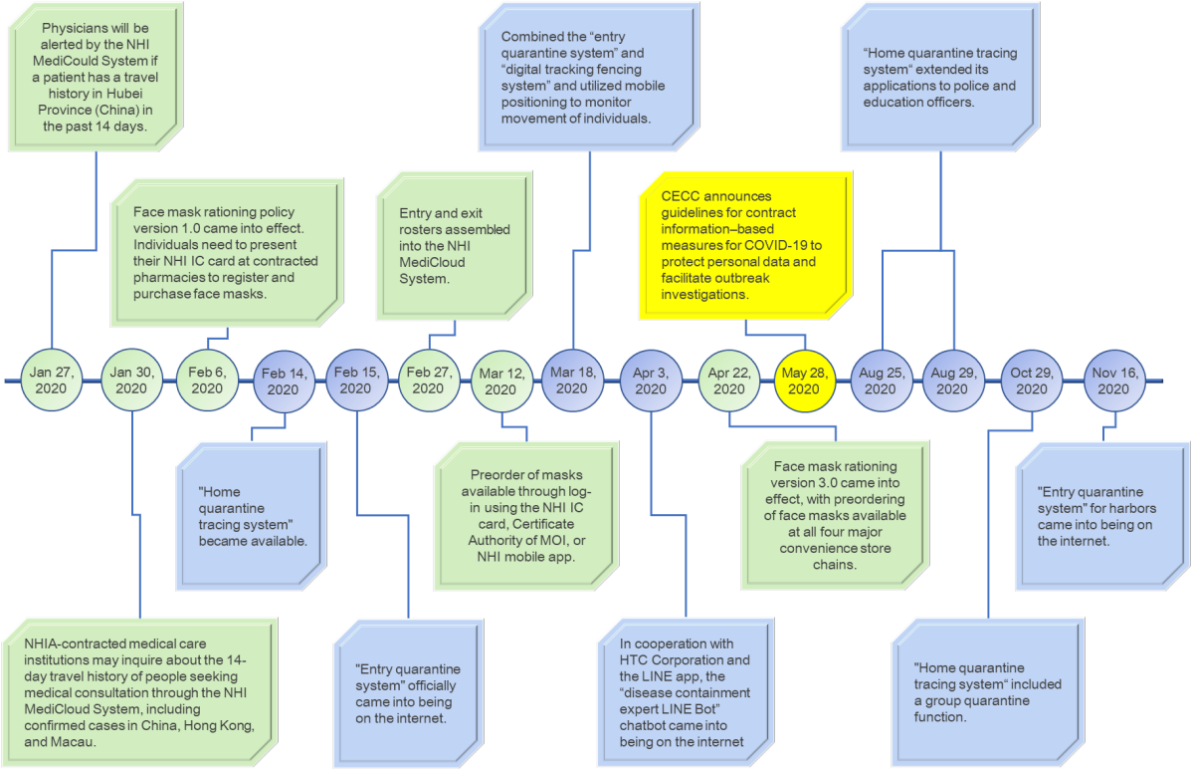
Timeline of technology usage in COVID-19 prevention in Taiwan. Green blocks represent the launching date of the travel history inquiry system and the name-based mask-rationing system, blue blocks represent the procedures and services for border control and the home quarantine system, and the yellow block represents the date when CECC announced guidelines for contact information–based measures and data collection. CECC: Central Epidemic Command Center, MOI: Ministry of the Interior, NHI: National health Insurance, NHIA: National Health Insurance Administration.

At the early stage of CECC establishment, the presence of fever, cough, as well as other respiratory symptoms, travel history, occupation, contact history, and cluster exposure were used as the criteria for case reporting and screening. In addition to the existing pandemic reporting and screening procedures, toward the end of January 2020 (also known as Lunar New Year for Asian-Pacific regions), the CECC decided to utilize the MediCloud system from NHIS to provide inquiry services for travel history. MediCloud system (previously known as “PharmaCloud”) had been expanding its services and functions to provide patient information (ie, disease diagnosis, examinations, laboratory testing, and medication) to hospitals and clinics since its initiation in 2015. After years of promotion and system advancement, the usage of MediCloud is currently over 99%, and MediCloud has become the essential platform for information sharing among medical institutions. Once interface linkage was established between the National Immigration Agency (part of the Ministry of the Interior of Taiwan) and the NHIA, the travel information database was integrated with medical records within just a few days. Now the NHIA has been able to provide inquiry services for travel history among infected countries or regions. In response to the progression of the COVID-19 pandemic, the features of querying wider ranges of travel history and occupations were included (Figure 2). These features contribute greatly to the prevention of nosocomial infections and enable timely screening to prevent COVID-19 outbreaks. Front-line medical workers are able to identify potentially infected individuals on the basis of their travel history and have reported symptoms with the aid of real-time alerts from the integrated information system and taken appropriate steps to adequately protect themselves from COVID-19.

### Name-Based Mask-Rationing System for Purchase

At the beginning of February 2020, the CECC announced that the daily production of face masks was approximately 2 million, which was markedly lesser than the total population of Taiwan (approximately 23 million). Meanwhile, China also proceeded with purchasing large amounts of face masks in response to the COVID-19 pandemic. Since the people of Taiwan have had experiences with the SARS pandemic, people in some areas started stocking up face masks at home to avoid shortage [9]. In response to the face mask shortfall, the government began to increase face mask production to ensure all citizens have equal access to high-quality face masks. The policy that the government deployed, the “name-based mask-rationing system,” inevitably ensured fair distribution of face masks in the community. Since all insurees (including legal foreign workers in Taiwan) obtain an NHI IC card, the CECC instructed the NHIS to contract pharmacies to link with the MediCloud system such that the policy of “three face masks per week per individual” can be executed for all insured individuals. The policy utilized the information that the NHI IC card provides to verify and control the allocation of face masks to ensure that everyone has access to face masks for basic protection against COVID-19. Furthermore, at the beginning of March, the CECC had announced a payment mechanism where the public can utilize kiosks to reserve face masks, pay the fees, and collect them at either local convenience stores or pharmacies per their preference. Local manufacturers in Taiwan have been able to increase face mask production; hence, the purchasing policy was modified by the CECC to “nine face masks every two weeks per individual” in early May. Starting on June 1, 2020, the public was allowed to make more purchases if needed, other than the primary rationing.

The current infrastructure of the NHIS allows the aforementioned functions such as travel history tracking and executing a “name-based mask-rationing system” policy; thus, all pandemic prevention strategies postulated by the CECC can be implemented in a timely manner. The establishment of the NHI IC card, MediCloud, and a virtual private network were mostly completed after the SARS outbreak in 2003. The original purpose was not pandemic control, but rather for Taiwan’s citizens to utilize medical services at their convenience. The original purpose of establishing the NHI IC card was to avoid the misuse of medical services by the public and to enhance medication safety by using information technologies to share information among hospitals, clinics, and pharmacies rather than for pandemic control. Meanwhile, this “centralized” data linkage structure has become the key enabler for the government to implement pandemic prevention strategies.

## Establishment of a Pandemic Prevention System from the Border to the Community

Toward the end of January 2020, the CECC decided to instruct passengers arriving from China, Hong Kong, and Macau to follow home quarantine procedures. The procedure required passengers to receive a home quarantine notice once they entered Taiwan (both by air and sea). Both the civil affairs department and the health department could be informed with lists of people who require home quarantine in their responsible areas. The civil affairs personnel would then monitor those who are in home quarantine with regard to basic living and health needs, and the health department would arrange for medical treatments if needed.

### Information-Driven System Planning

Owing to the lack of assistance from DT, the manpower required to carry out those surveillance duties was tremendous and ineffective in the beginning. For instance, the airport staff needed to identify those who require home quarantine from among 70,000 passengers entering Taiwan each day. Therefore, airports needed to recruit a large number of health officers or temporary assistants to carry out verification duties. According to the infectious disease prevention and treatment law, the duty of performing home quarantine is fulfilled by civil affairs personnel of the Ministry of Home Affairs and the local government. Health officers who are responsible for COVID-19 testing at the airport collected all the information on paper, performed web-based data entry, and the information was sent out from the health department to the Ministry of Home Affairs and then passed on to the local government. The local government passed on the information regarding the results of COVID-19 testing to the chief of the village, who in turn would check and assist those who are in home quarantine. Those who violate home quarantine procedures are subject to administrative penalties by the health administrative system. Many government employees are under extreme pressure with regard to mishandling of COVID-19–related tasks and the creation of loopholes in the disease prevention system during the pandemic. The delay in information received by the local government employees, misinformation regarding personal data (such as telephone numbers and addresses), quality of information, and efficiency may all contribute to the pressure felt by the government employees.

By the end of January 2020, the CECC had begun to improve data quality and efficiency and attempted to improve the sharing of correct personal information such as residential addresses, telephone numbers, cellphone numbers, and the start date of home quarantine among different government departments. As an information center, the CECC has established both the information flow and a relevant application system to allow for the circulation of information from the border to the community. Because 80% of the people of Taiwan are smartphone users, smartphones were being used as the main tool to keep track of individuals. A mobile app was not considered a viable option regardless of the smartphone’s operating system (iOS or Android) since it may take considerable time for approval. Nevertheless, more time may be required for program correction. Therefore, the option of using a mobile app was eliminated as it was time-consuming during the critical outbreak.

### Establishment of the Information-Driven System

**Figure 3:**
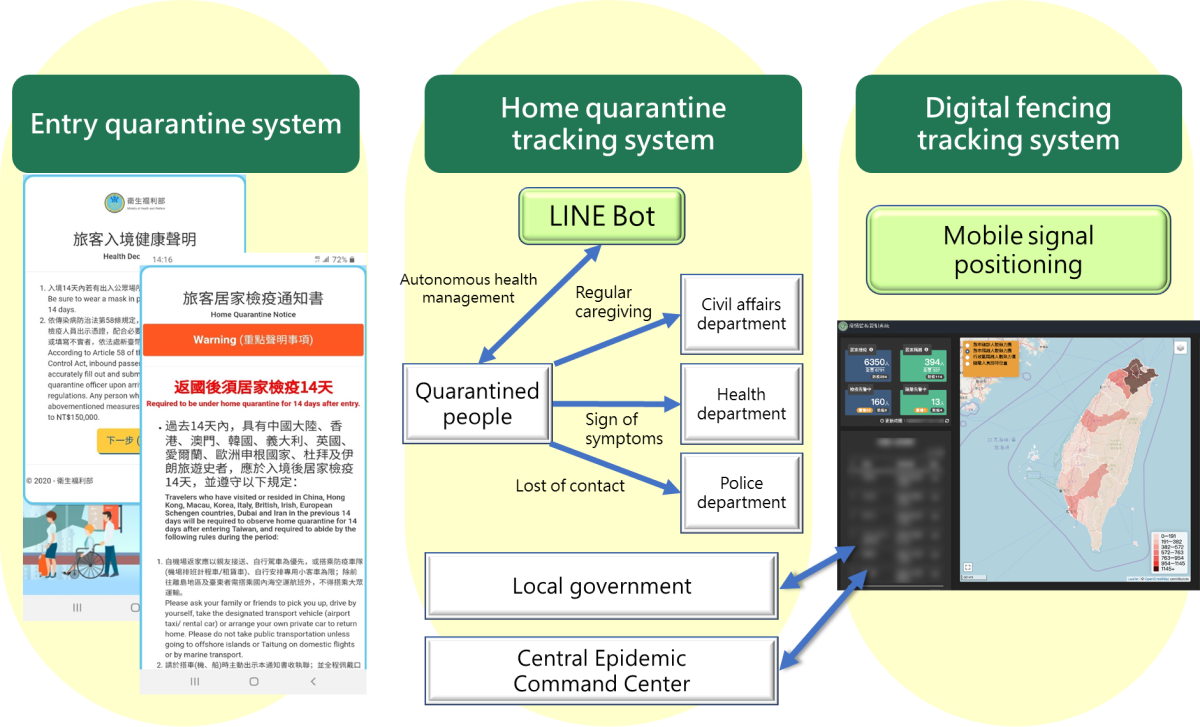
Diagrammatic representation of the border control and home quarantine systems.

The information-driven system was ready for launch after 2 weeks of brainstorming ideas to the completion of the entire system. In mid-February 2020, the information system became available and allowed data linkage among border control and local government services with the Advance Passenger Information System from the National Immigration Agency and Household Registration Information from the department of household affairs (also part of the Ministry of the Interior of Taiwan) for the management of home quarantine cases (Figure 3). For instance, the information system would ask all inbound passengers to use smartphones to scan a specific QR code prior to their departure and fill out personal information, health status, travel history, cellphone number, and public transportation needs. Before the aircraft reaches the gate, passengers will receive text messages regarding information on home quarantine notice and health declaration certificates. If passengers report symptoms related to COVID-19 under health status, the health personnel would proceed to test them immediately and arrange for medical treatment. If passengers indicate the need for public transportation, arrangements would be made. At present, there are on average 40,000 travelers arriving in Taiwan every day, and our information system has been functioning well in entry control.

Immigration information can be delivered in a timely manner to the home quarantine management system, which is based on the regional joint defense concept to directly assign people who require home quarantine to the village, which prevents delays in information transfer. Initially, when the system was established, issues such as the submission of incorrect contact information were reported. The village-based civil affairs personnel (such as village officials) are responsible for confirming whether the individual has returned to the correct home quarantine location; if not, all the other possible addresses or telephone numbers may be used to track the individual down. If needed, the civil affairs personnel would check on the individual in person to ensure the individual stays at the designated quarantine location. The civil affairs personnel from different villages would also assist one another to carry out the surveillance duty. During the 14-day home quarantine period, the civil affairs personnel would provide home care services to the quarantined individuals on the basis of the address and contact number that appears in the system. Home care services provided by the civil affairs personnel are often delivered to the individual through smartphones, which will allow tracing of records for future reference. If there is an incidence of violation of home quarantine, the CECC would coordinate with telephone service providers to send out timely text messages to remind the concerned individual to return to their designated home quarantine location.

### Merging of Information-Driven Systems and Instant Messaging Apps

**Figure 4:**
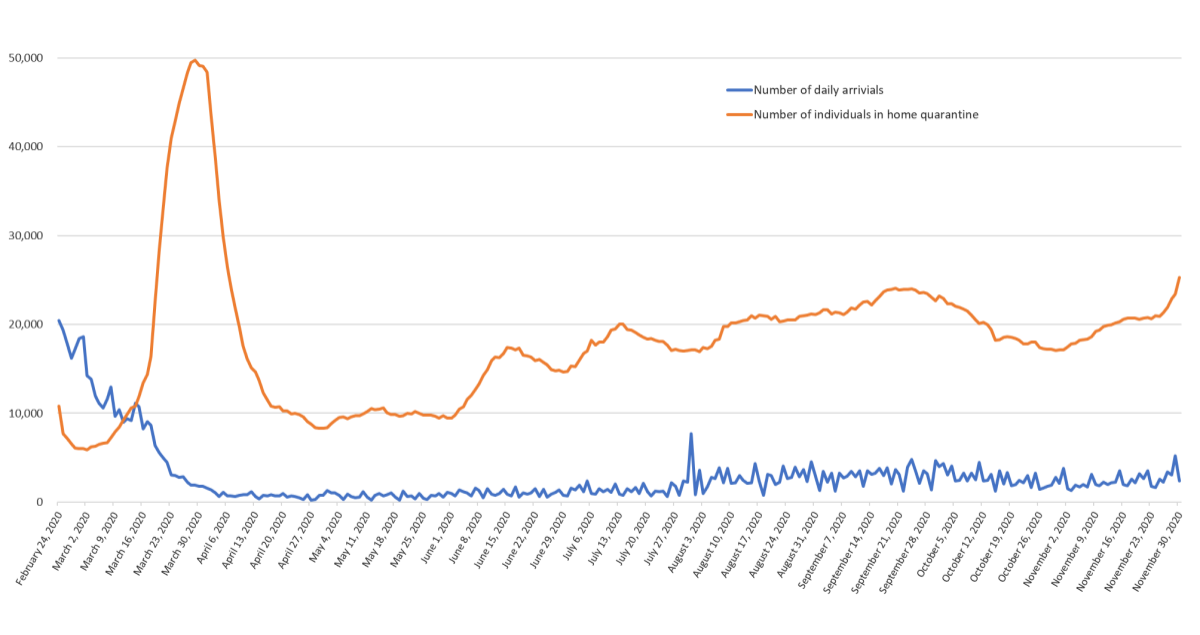
Number of daily arrivals and home quarantine management in Taiwan (data were collected until November 30, 2020).

As the system functions better each day and the accuracy of information continues to improve, the initiation of complete border control still remains a challenge. On March 19, 2020, the CECC announced that all travelers entering Taiwan will need to fulfill the home quarantine requirement. Although on average only approximately 7000 travelers entered Taiwan each day, the number of home quarantine cases still increased significantly (maximum 50,000 people/day to be monitored), and this certainly adds tremendous pressure on the local civil affairs personnel (Figure 4). The CECC used the media as a channel to advocate and invite the citizens to use the instant messaging app LINE and add the LINE Bot as a friend. On obtaining a consensus from home quarantine cases, the local civil affairs personnel could use the LINE app to follow up on home-quarantined individuals with regard to self-health management and personal needs on a daily basis. According to a survey performed in mid-May 2020, 7100 home-quarantined people in Taiwan who used the LINE app were sampled. The data revealed that chatting with LINE Bot and chatting with local civil affairs personnel scored 8.68 and 8.56, respectively, on a scale of 10 (0=completely unsatisfactory and 10=very satisfied).

Until June 30, 2020, form among 447 confirmed cases of COVID-19, 356 (80%) were imported, and only 55 (12%) local cases and 36 (8%) confirmed cases were from navy clusters. For the imported cases, 141 infected individuals tested positive on arrival at the Taiwan border, 150 infected individuals tested positive during the 14-day quarantine, and the remaining 65 individuals tested positive during cluster quarantine, home quarantine, or isolation (Table 1). The information-based disease control system was the main “driver” to successfully block the spread of COVID-19 at the border and away from the community. The local rate of penalization for violating the home quarantine policy was lower than 0.5% (n=720/175,720 people).

**Table 1:** Source of confirmed COVID-19 cases in Taiwan as on November 30, 2020.

Records			Quantity
Tests performed, n			110,685
**Confirmed cases (positive rate), n (%)^a^**			679 (0.6)
	**Imported cases**		588 (86.6)
		Immigration testing	232 (39.5)
		During home quarantine	207 (35.2)
		Others	149 (25.3)
	Locally acquired infections		55 (8.1)
	Navy crew members aboard the Panshi Fast Combat Support Ship		36 (5.3)

^a^Imported cases, locally acquired infections, and navy crew members aboard the Panshi Fast Combat Support Ship are calculated relative to the number of confirmed cases.

## Recommendation and Discussion

From the perspective of using data linkage to enhance pandemic control, the success of COVID-19 control in Taiwan was analyzed and could be attributed to 2 main data linkage models. These 2 main models enhance and improve 4 key public health strategies in Taiwan, including border control, prevention of community outbreaks, enhancement of personal hygiene, and prevention of nosocomial infections. Through data linkage between the NHIS and other departments, Taiwan was able to secure the supply of face masks and avoid nosocomial infections, which have been reported in previous studies [10,11]. The efficiency of the “centralized” data linkage model can be optimized as long as the integrity of the database is sufficient. Moreover, the range of travel history and the traveler’s identity can be expanded as the pandemic progresses. The amount of allowance to purchase face masks and participating vendors can also be adjusted as needed. Overall, it is quite feasible to have the aforementioned flexibilities in a country such as Taiwan where the NHIS has demonstrated great functionality and performance. Unlike Taiwan, the United States has multiple insurance systems. Nevertheless, it is still feasible to use existing web-based medical networks to create similar data linkages through additional effort. From the border to the community, the information-oriented prevention system is considered a type of “downstream supply chain” of data linkage, and the key to its success is attributed to the accuracy and timeliness of data collection [6,12]. For instance, for people who require home quarantine, within the first hour or 2 hours of arrival, the information should be sent directly to the civil affairs personnel of the appropriate residential area. This process can significantly ensure that information is received in a timely manner and can avoid any flaws in disease prevention. It is essential to implement the correct system at the right time to reinforce border management and prevent community outbreaks. Therefore, using the agile approach instead of the traditional approach in building the correct system is preferable during the COVID-19 outbreak.

Similar to other countries that rely heavily on information technologies, it is essential to establish a balance between disease prevention and personal data protection. However, the public generally agrees with and follows the necessary pandemic control policies despite the concern of personal data protection. Unlike Taiwan, South Korea has implemented a law allowing the government to reduce the level of personal data protection during the critical phase of the pandemic [13]. The government of Taiwan applies the current Infectious Disease Control Act and Personal Data Protection Act and openly discloses the prevention measures that are required to be followed by all travelers on entering Taiwan. On May 28, 2020, the CECC announced guidelines for contact information–based measures and data collection in response to the COVID-19 outbreak to ensure personal data protection and facilitate outbreak investigations. On the other hand, Taiwan chose to use a less privacy-sensitive DT approach, such as using smartphone-based station tracking instead of real-time GPS technology to monitor people who are in home quarantine or home isolation. Furthermore, a proposal has been approved, endorsed, and authorized by the legislature to eliminate all the unnecessary electronic records and linkages after the pandemic, which can also be adopted by other countries.

Currently, Taiwan has been free from local COVID-19 transmission for more than 200 days. The CECC has cautiously reopened the economy in a stepwise manner. As the restrictions on social activities are being lifted gradually, the “from border to community” information-driven prevention system continues to strengthen border control, ensuring that imported cases can be isolated immediately to minimize contact with others and information on the number of screenings performed and the number of confirmed cases can be compiled at the airport on a daily basis. Furthermore, the system can also help analyze and understand the changes in the number of confirmed cases overseas once border restrictions are lifted and adjustments are made to border control policies accordingly. Furthermore, in the post–COVID-19 era, new technology-driven practices will now form a part of “the new normal.” The government and organizations need to adopt new DT systems during the pandemic and devise methods to normalize these new practices. These new technology-driven work practices are usually implemented during the most severe times under high-pressure conditions and often without former experience or training. Taiwan’s experience can therefore be considered a valuable reference for other countries to further the current understanding of how DT can be embedded within government practices and form the “new normal” in the post–COVID-19 era.

In conclusion, the integrity, accuracy, and timeliness of data linkage and DT infrastructure ensure that essential public health interventions, such as border control, quarantine, case detection, contact tracing, and universal surgical mask–wearing can be effectively implemented and become the foundation of the highly successful COVID-19 pandemic response in Taiwan.

## Abbreviations

CECC: Central Epidemic Command Center
DT: digital technology
NHIA: National Health Insurance Administration
NHI IC card: National Health Insurance IC card
NHIS: National Health Insurance System
SARS: severe acute respiratory syndrome
